# Lithium can mildly increase health during ageing but not lifespan in mice

**DOI:** 10.1111/acel.13479

**Published:** 2021-09-17

**Authors:** Tobias Nespital, Brit Neuhaus, Andrea Mesaros, André Pahl, Linda Partridge

**Affiliations:** ^1^ Max‐Planck Institute for Biology of Ageing Cologne Germany; ^2^ Institute of Healthy Ageing, and GEE, UCL London UK; ^3^ Present address: medproduction GmbH Cologne Germany

**Keywords:** ageing, healthspan, lifespan, lithium

## Abstract

Lithium is a nutritional trace element, used clinically as an anti‐depressant. Preclinically, lithium has neuroprotective effects in invertebrates and mice, and it can also extend lifespan in fission yeast, *C. elegans* and *Drosophila*. An inverse correlation of human mortality with the concentration of lithium in tap water suggests a possible, evolutionarily conserved mechanism mediating longevity. Here, we assessed the effects of lithium treatment on lifespan and ageing parameters in mice. Lithium has a narrow therapeutic dose range, and overdosing can severely affect organ health. Within the tolerable dosing range, we saw some mildly positive effects of lithium on health span but not on lifespan.

## Graphical abstract

Lithium chloride and carbonate administrated with the chow to mice in several doses did not extend lifespan, but mildly improved motor performance, body weight and composition, glucose tolerance, and kidney pathology during ageing in males.

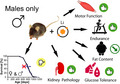

The anti‐depressant and mood stabilizing effects of lithium were discovered the mid 20th century (Cade, 1949, Schou et al., 1954), and administration of lithium salts is still the first‐line therapy for bipolar disorders (Alda, 2015). Lithium can also ameliorate pathology in animal models of neurodegeneration (Partridge et al., 2020), through multiple molecular mechanisms, and has been proposed as a therapy for Alzheimer's Disease (Damri et al., 2020). Suggesting that it may have a broader therapeutic range, lithium can also extend lifespan in fission yeast (Sofola‐Adesakin et al., 2014), *C. elegans* (McColl et al., 2008; Tam et al., 2014) and *Drosophila* (Castillo‐Quan et al., 2016 and 2019), in the last by inhibition of GSK‐3 and activation of the transcription factor NRF2. Human survival across 18 Japanese municipalities correlated with increased lithium level in drinking water (Zarse et al., 2011). These findings suggest that conserved molecular responses to lithium treatment could improve health during ageing in mammals (Partridge et al., 2020). In this study, we therefore analysed the influence of lithium treatment on lifespan and parameters of health during ageing in mice.

To determine the concentration of lithium suitable to be administered in a longitudinal ageing study, we first tested the effects of lithium chloride (LiCl) in doses from 0.01 to 2.79 g LiCl) per kg chow, which includes the dosing range in published mouse studies (Fiorentini et al., 2010, Noble et al., 2005, Tajes et al., 2009, Kitazawa et al., 2008, Gomez‐Sintes and Lucas, 2010). C57Bl/6J mice fed with 1.05–2.79 g/kg LiCL in the diet showed lithium plasma levels between 0.4 and 0.8 mM/l. We assessed the effect of lithium on serine‐9 phosphorylation of hippocampal GSK‐3β, which was significantly increased for all doses down to 0.1 g LiCl/kg (Figure S1). While plasma levels to 0.4 and 0.8 mM/l are well tolerated by human patients, at doses above 1.44 g LiCl/kg, we observed an obvious dose‐dependent polydipsia combined with a distinct polyuria, pointing towards a significant degree of kidney toxicity. Similarly, a washout effect caused by highly increased drinking behaviour was observed when male and female mice of the same C57Bl/6J strain were treated with 0.64 g LiCl/kg from 19 months of age (Evans et al., 2021). For the doses from 1.05 to 1.44 g LiCl/kg, we additionally observed dose‐dependent focal karyomegaly characterized by enlarged nuclei in the renal tubular epithelium, a form of kidney toxicity. The focal karyomegaly was absent from mice receiving lower lithium doses (0.01 – 0.1 g/kg diet) and also from control mice.

We therefore carried out life‐long lithium treatment in the range from 0.02 to 1.05 g/kg diet. Administration to both sexes at doses of 0.02 and 0.05 g/kg starting at 3 months or 18 months of age did not affect lifespan (Figure 1b, Figure S2). In an additional group, treatment of females with 0.1 g/kg starting at 19 months of age also had no significant effect (Figure S3). Treatment of male and female mice from an age of 3 months with 0.02 and 0.05 g/kg LiCl, and then switching late in life at 22 months to 0.5 and 1.05 g/kg, respectively, had no effect on male survival and reduced maximum lifespan of females (survival of last 20% of animals to die), (Figure 1c).

**FIGURE 1:**
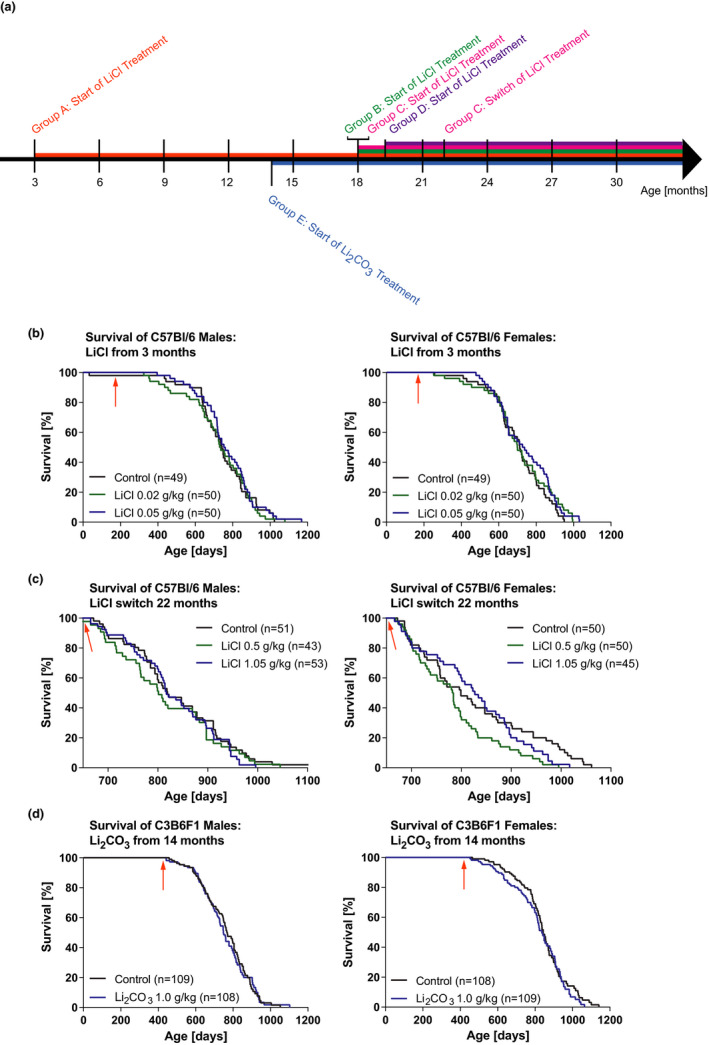
Effects of lithium administration at varying doses on lifespan. (a) Schematic overview of the different treatment regimens. Group A: ♀ & ♂, *n* = 49–50 per treatment; Group B: ♀ & ♂, *n* = 50 per treatment; Group C: ♀ & ♂, *n* = 43–53 per treatment; Group D: ♂, *n* = 43 per treatment; Group E: ♀ & ♂, *n* = 108–109 per treatment. (b) Survival curves for male and female C57Bl/6J mice exposed to LiCl from 3 months of age (see arrow, *n* = 49–50). (c) Survival curves for male and female C57Bl/6J mice exposed to 0.02 and 0.05 g/kg LiCl from 18 months of age and then switched at 22 months (see arrow) to the indicated doses (female maximum lifespan < 20% survival: *p* = 0.0003, *n* = 43–53). (d) Survival curves for male and female C3B6F1 mice exposed to Li_2_CO_3_ from 14 months of age (see arrow, *n* = 109). Statistical analyses by log‐rank test

Different mouse strains can respond differently to drugs, and we therefore assessed the effect of lithium on lifespan in C3B6F1 mice (F1 hybrids of C3H/HeOuJ females and C57Bl/6N males). Lithium can also be administered either as the chloride or the carbonate, which is mostly used in higher concentrations than the chloride without causing harmful side effects at 2 to 2.4 g/kg (Choi et al., 2011; Contestabile et al., 2013; Kim et al., 2013). In a pre‐experiment, we used a concentration range well below these doses and determined an optimal dose of 1.0 g/kg Li_2_CO_3_ as the highest dose that inhibited GSK‐3β and did not induce increased drinking (Figure S4) to assess lifespan and healthspan. Lifelong treatment at this dose starting at 14 months of age and also did not extend lifespan of either sex (Figure 1d). Administration of 0.64 g Li_2_CO_3_/kg from 19 months of age to male and female C57Bl/6J mice has also been reported to have no effect on mouse survival (Evans et al., 2021).

We assessed the effects of lithium on other age‐related phenotypes of the mice. During the first year of their life, doses of 0.02 and 0.05 g/kg LiCl administered from 3 months of age significantly decreased the growth of male mice, due to reduced fat content (Figure 2b), while females were not affected (Figure S5a). Old male mice (26–28 months) that were treated from an age of 18 months with 0.02 and 0.05 and then switched at 22 months to 0.5 and 1.05 g/kg, respectively, had a dose‐dependent lower body weight and fat content compared to age‐matched controls (Figure 2d), while females were less affected (Figure S5d). The reduced fat content in old males compared to midlife (Figure 2d vs. 2b) may be explained by their more advanced ageing process given that they have considerably shorter lifespans than females (Figure S5b vs. a). The decreased fat mass despite unaffected food consumption indicates an effect of lithium on lipid metabolism. Mice on the low doses of 0.02 and 0.05 g/kg LiCl administered from 3 months of age showed delayed age‐related loss of glucose tolerance (Figure 2c). In addition, male mice that were switched to 0.5 and 1.05 g/kg at 22 months, after being treated with 0.02 and 0.05 g/kg from an age of 18 months, respectively, showed significantly increased tolerance to glucose at ages over 26 months (Figure 2e). Neither treatment improved glucose tolerance in females (Figure S6).

**FIGURE 2:**
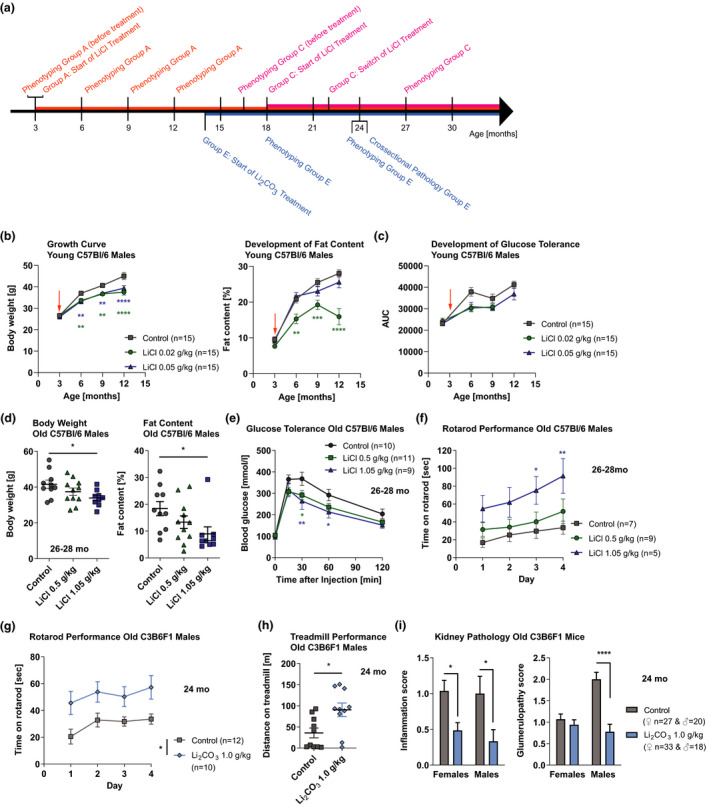
Effects of lithium administration at varying doses on age‐related phenotypes of mice. (a) Schematic overview of the different treatment regimens. Group A: ♀ & ♂, *n* = 15 per treatment; Group C: ♀ & ♂, *n* = 9–11 per treatment; Group E: ♀ & ♂, *n* = 10–15 per treatment. (b) Growth curve (*p* = 0.0001, n = 10–29) and development of fat content (*p* < 0.0001, n = 10–15), and (c) glucose tolerance (*p* = 0.018, n = 9–15) of C57Bl/6J males exposed to LiCl from 3 months of age (see arrow) during their first year of life. (d) Body weight (*p* = 0.047, *n* = 9–11) and fat content (*p* = 0.0507, n = 9–11), (e) glucose tolerance (*p* = 0.049, *n* = 7–10) and (f) rotarod performance (*p* = 0.047, *n* = 5–9) of C57Bl/6J males exposed to LiCl from 22 months of age at age of 26–28 months (26 mo). (g) Rotarod (*p* = 0.0101, *n* = 10–12) and (h) treadmill (*p* = 0.013, *n* = 10) performance of C3B6F1 males, and (i) inflammation (*p* = 0.0003, *n* = 18–20) and glumerulopathy (*p* < 0.0001, *n* = 18–20) score in the kidney of C3B6F1 mice of both sexes exposed to Li_2_CO_3_ from 14 months of age at age of 24 months (24 mo). Error bars indicate SEM. Statistical analyses were performed using the restricted maximum likelihood method in a mixed‐effects model for (b) and (c), one‐way ANOVA for (d), two‐way ANOVA for (e), (f), (g) and (i), two‐tailed unpaired t test for (h), and Dunnett's or Tukey's multiple comparisons test for (b), (c), (e), (f), (g) and (i). *p < 0.05, **p < 0.01, ***p < 0.001, ****p < 0.0001

There was a dose‐dependent increase in motor function on the rotarod in old males under LiCl treatment (Figure 2f), possibly related to their lower body weight, while similar trends in forelimb grip strength or treadmill were not significant (Figure S7). Additionally, in 24‐month‐old Li_2_CO_3_‐treated mice, both motor function on the rotarod and endurance on the treadmill were significantly increased in males (Figure 2g and h), with no effect in females (Figure S7a and S8). Administration of 0.64 g Li_2_CO_3_/kg from 19 months of age on to 120 mice did not increase metabolic and activity phenotypes (Evans et al., 2021). However, we found that, at least in males, lithium can positively affect health at old age.

Histopathological analysis of 2‐year‐old, Li_2_CO_3_‐treated, C3B6F1 mice showed reduced age‐related pathologies in the kidneys, with significantly decreased kidney inflammation (leukocyte infiltration) in both sexes, which in males coincided strongly with a reduction of glomerulopathy (Figure 2i). This is interesting, because lithium commonly causes renal side effects in humans, such as polyuria, proteinuria and reduction in glomerular filtration rate (Gong et al., 2016; Grünfeld & Rossier, 2009; Xu et al., 2014). However, the antiproteinuric mode of action of lithium reduces glomerulosclerosis in mice with nephropathies (Xu et al., 2014) and protects the glomeruli in experimental models of glomerular disease, such as the NZB/W mouse with a spontaneous lupus‐like autoimmune disease (Lenz et al., 1995), and reduces renal inflammation through GSK‐3 inhibition (Wang et al., 2009). These direct kidney protective effects of lithium (Gong et al., 2016) may explain our results, which may have resulted from the low doses of lithium used. Other organs seemed to be largely unaffected.

Considering the use of a broad range of well‐tolerated lithium concentrations, different lithium salts and different mouse strains, we conclude that, in contrast to the findings in yeast, worms and flies, lithium does not seem to be a promising candidate for geroprotection in humans. Although it caused mild improvements in body weight and composition, glucose tolerance and motor performance, these were largely confined to males and were not accompanied in either sex by increased lifespan. Further work on the mechanisms underlying the geroprotective effects of lithium in invertebrates may reveal more specific targets for intervention in mammals.

## CONFLICT OF INTEREST

None declared.

## AUTHOR CONTRIBUTIONS

Conceived of the study: LP, BN, TN; participated in its design and coordination: TN, LP, BN, AM; carried out experiments: TN, AP, BN; drafted the manuscript TN, LP.

## Supporting information

Supplementary Material

## Data Availability

The data that supports the findings of this study are available in the supplementary material of this article.
