# Supplementary material for: Lithium can mildly increase health during ageing but not lifespan in mice
Source: Aging Cell. 2021 Sep 17;20(10):e13479. doi: 10.1111/acel.13479 (PMC8520709; doi:10.1111/acel.13479)
Supplement: Supplementary file 1 — Supplementary Material [file ACEL-20-e13479-s001.docx]

**Online Supporting Information**

**Experimental Procedures**

**Mice and lithium treatment**

C57Bl/6J WT mice for lifespan and phenotypic analysis were generated from C57Bl/6J WT parents derived from our in-house C57Bl/6J breeding colony. Mice in this colony were originally derived from Charles River Germany, and maintained by inbreeding for a maximum of 4 generations before being substituted by a fresh Charles River-derived breeding colony. C3B6F1 hybrids were also bred in-house by a cross between C3H/HeN female and C57Bl/6N male mice, which were obtained from Charles River Laboratories. Directly after birth, litters with more than 8 pups were reduced to a maximum of 8 pups, while litters with fewer than 4 pups were excluded from the analysis to avoid lifespan-modulating effects of mal- or overfeeding during the nursing period. All lifespan and phenotyping mice were weaned at 3 weeks of age. While females were randomized upon weaning, male mice were weaned litterwise to avoid aggression and fighting. If males of different litters had to be combined, a ratio of 2:3 was preferred over a 4:1 ratio. Lifespan analysis and phenotyping were performed on different cohorts of mice. All mice were housed in individually ventilated cages, in groups of five mice per cage, under specific-pathogen-free conditions, at 21°C, with 50-60% humidity and 12h light/dark cycle. Mice had ad libitum access to chow (Ssniff Spezialdiäten GmbH; 9% fat, 34% protein, 57% carbohydrates) and drinking water at all times, unless withdrawal of chow was necessary for specific experiments within the phenotyping cohort. Mice received a standard rodent diet (Ssniff, R/MH, low phytoestrogen content) for their lifetime (placebo group) or were switched to one of two different lithium-enriched diets (Ssniff, R/MH low phytoestrogen content, supplemented with LiCl or Li_2_CO_3_, respectively). Experiments were conducted under the approval of the State Office for Nature, Environment and Consumer Protection North Rhine-Westphalia (approval no. 84-02.04.2011.A120 and 84-02.04.2017.A074).

**Mouse pre-experiment**

Mice for the LiCl dosage pre-experiment were received from Charles River, Germany. During the pre-experiment, 10 male and 10 female mice at the age of three months were fed for six weeks with one of ten different diets, including a placebo diet without lithium substitution and nine LiCl-enriched diets (0.01 g/kg diet, 0.05 g/kg diet, 0.1 g/kg diet, 1.05 g/kg diet, 1.44 g/kg diet, 1.74 g/kg diet, 2.1 g/kg diet, 2.44 g/kg diet and 2.79 g/kg diet). During the 6-week observation period, food consumption and body weight were determined weekly. At the end of the test phase, blood samples were taken to determine lithium plasma levels. Mice were killed and kidneys were removed for a pathological assessment. To determine the Li_2_CO_3_ dose for the longevity study, during another pre-experiment, 10 male and 10 female mice at the age of ten months were fed for eight weeks with one of four different diets, including a placebo diet without lithium substitution and three Li_2_CO_3_-enriched diets (0.5 g/kg diet, 1.0 g/kg diet and 1.5 g/kg diet). During the 8-week observation period food and drinking water consumption and body weight were determined weekly. At the end of the test phase, mice were killed and tissues were collected.

**Mouse lifespan**

Survival under lithium chloride (LiCl) treatment was assessed from a total of 600 C57Bl/6J mice, half males and half females, which were bred in two generations from the same breeding pairs. Mice were subdivided into two cohorts of 300 mice each, half males and half females. In the first cohort, dietary switch was done at 3 months of age, in the second cohort at 1.5 years of age. Within each cohort, 50 males and 50 females were switched to the lower lithium diet containing 0.02 g LiCl /kg diet, while another 50 males and 50 females were switched to the higher dose lithium diet with 0.05 g LiCl /kg diet. The remaining 50 males and 50 females continued to receive the placebo diet without lithium. After finding no effect of lithium treatment on lifespan in these cohorts, we used a cohort of 300 C57Bl/6J mice, half males and half females, which were treated from an age of 18 months of age with 0.02 and 0.05 g LiCl /kg diet to be switched at 22 months of age to the increased LiCl concentrations of 0.5 and 1.05 g/kg diet, respectively, to assess their lifespan.

Survival under lithium carbonate (Li_2_CO_3_) treatment was assessed from a total of 436 C3B6F1 mice, half males and half females, which were bred in three generations from the same breeding pairs. Mice were subdivided into two cohorts of 218 mice each, half males and half females, of which half was administered 1.0 g Li_2_CO_3_ /kg diet from 14 months of age on while the other half received the control diet without any added lithium. 212 mice were used purely for lifespan analysis, 120 mice were used for the phenotyping experiments and the other 104 mice for pathological analysis at old age. All mice of the latter two groups, which survived until 24 months of age, were used for tissue collection to assess organ pathology. For lifespan analysis, these mice were then censored.

All mice were monitored for health issues daily, lifespan and tissue collection mice were otherwise left undisturbed until they died, phenotyping mice were used for the experiments at the planned time points. Sick mice were kept under tight control and weighed daily. Health status of severely diseased mice was assessed according to a strict protocol in concord with the applicable animal welfare rules and the ethical committee guidelines. If death became imminent, mice were killed and inspected for gross pathological changes.

Kaplan-Meier survival curves were generated using the birth and death dates of each individual mouse. Differences between groups were evaluated using the log-rank test. Maximum and minimum lifespan were calculated from the oldest and youngest 20% of mice in each treatment group.

**Mouse phenotyping**

Phenotyping was performed on separate cohorts of mice. A total of 720 C57Bl/6J mice, half males, half females, were bred in 3 generations. Assignment to the different treatment groups and timing of the dietary switches were equivalent to the lifespan cohorts, however with 60 males and females per group instead of 50. Experiments were performed from the age of three months onwards. For the mice fed with LiCl at a young age, most experiments were repeated at quarterly intervals up to 12 months of age. For the mice receiving the lithium diet from 18 months of age and then switched to the increased LiCl concentrations at 22 months of age (0.5 and 1.05 g/kg diet), experiments were done at 3, 16-17 and 26-28 months of age. To reduce stress levels, the 60 female and 60 male mice in each treatment group were subdivided in subgroups of four times 15 mice, which were either used for glucose homeostasis tests, motor performance tests, blood sampling or CT/MRT measurements. For the 120 C3B6F1 mice, half males and half females, of which half was administered with 1.0 g Li_2_CO_3_ /kg diet from 14 months of age on and the other half received the control diet without any lithium, experiments were done at 18 and 24 months of age. To reduce stress levels, the 30 female and 30 male mice in each treatment group were subdivided in subgroups of twice 15 mice for different sets of experiments.

All mouse procedures were conducted in accordance with European, national and institutional guidelines and were approved by local government authorities (approval no. 84-02.04.2011.A120 and 84-02.04.2017.A074).

**Drinking water consumption measurement**

Drinking water consumption was measured in the lithium carbonate pre-experiment weekly. For drinking water consumption measurements, the drinking water bottle was weighed when placed into the holding cage and again when replaced. Average drinking water consumption per mouse per day was determined from the weight difference.

**Glucose tolerance test**

For glucose tolerance tests mice were starved for 16 hours. Body weight and fasted blood glucose levels were determined, before each mouse received an intraperitoneal injection of 20% glucose (DeltaSelect, solution for infusion, 10 ml/kg body weight). Blood glucose levels were determined 15, 30, 60 and 120 minutes after glucose injections.

**Rotarod analysis**

Rotarod analysis was performed to test for differences in overall fitness and coordination. Mice were placed onto the Rotarod (TSE Systems, type 3375-M5) as it was rotating at a low speed (5 rpm). After starting the measurement, rate of revolution was continuously increased from 5 rpm to 40 rpm over a total period of 300 seconds. We measured the length of time that each mouse spent on the rod, with a cut-off time of 300 seconds. The test was performed two consecutive times for each animal and on four consecutive days.

**Treadmill**

Treadmill exercise was performed to test mice for differences in their overall fitness (TSE Systems, type 3033401-M-04/C). Mice were placed onto the slowly moving belt (0.1m/sec) and allowed to warm up and get used to the new environment for 5 minutes. Afterwards, measurement was started and mice were run to exhaustion, running at a speed of 0.1 m/sec for another ten minutes, before speed was constantly increased to 1.3 m/s within 60 min. To ensure that mice did only stop running upon exhaustion, an electric grid at the end of the belt driven by a light barrier produced a weak stimulus (0.3 mA) as soon as mice slowed down beyond a critical point and crossed the laser beam for more than 2 seconds. Length of the stimulus was set to 5 seconds followed by a 5 second refractory period. Exhaustion was defined as the willingness of a mouse to sustain three consecutive shocks instead of returning to the running belt.

**Grip strength**

The grip strength test is a non-invasive method to evaluate mouse limb strength. We used an automatic grip strength meter to assess mouse forelimb strength. Mice were suspended by their tail, were held in front of the metal bar connected to the power amplifier and pulled back as soon as they gripped the metal bar. Each mouse was tested 5 times in a row. Whenever a mouse happened to grip the bar with fore- and hind limbs or whenever a mouse refused to pull the bar properly but released it immediately the measurement was not evaluated and repeated.

**Body composition**

Body fat content was measured by *in vivo* nuclear magnetic resonance using a minispec mq 7.5MHz (Mq7.5 NMR Analyzer, Bruker optics)

**Blood/Plasma sampling**

Blood samples were taken by incision of the tail vein. About 100 µl of blood were collected and spun down for 15 minutes at 4°C and 13.000 rpm. The plasma was transferred to a fresh tube and stored at -20°C. Blood samples for the determination of lithium plasma levels during the pre-experiment.

**Tissue sampling**

Tissues for biochemical analysis were harvested, shock-frozen in liquid nitrogen and stored at -80°C. Kidneys for mouse kidney pathological screening (LiCl pre-experiment) were dissected, put into 4% formaldehyde and shipped to Charles River Edinburgh. Tissues for cross sectional pathology were harvested, fixed in 4% formaldehyde and shipped to Prof. Robert Klopfleisch (Institute of Veterinary Pathology, Freie Universitaet Berlin) for pathological assessment.

**Determination of lithium plasma levels**

Lithium plasma levels were determined via flame emission spectrometry at the university hospital Cologne in the institute of clinical chemistry and partly via a lithium-ion sensitive electrode (EasyLyte electrolyte analyzer, Medica Corporation, Bedford, USA) in our own institute.

**Generation of hippocampal lysates and western blot analysis**

The hippocampus was dissected from frozen mouse hemispheres and lysed in 200 µl ice-cold lysis buffer (10 mM Tris-HCl pH 7.4, 0.32M sucrose, 800 mM NaCl, 1 mM EGTA and 1 Roche cOmplete mini pill EDTA-free per 10 ml buffer). The tissue was sonicated 15 times at 30% amplitude on ice. Protein concentration in the samples was measured with the Pierce BCA System according to the manufacturer’s protocol. Lysates (20 µg protein) were resolved by SDS-PAGE, transferred to nitrocellulose membranes and immunoblotted with appropriate antibodies according to the manufacturer’s protocols. Quantification of signals was done with the ImageJ software (NIH, Bethesda, USA) and the GraphPad Prism software (GraphPad Software, La Jolla, USA). **Antibodies**: anti pSer9-GSK3beta antibody (#9336) was purchased from Cell Signaling Technologies (Beverly, MA), total GSK3 alpha/beta (sc-7291) and anti GAPDH antibody (sc-25778) were supplied by Santa Cruz Biotechnology (Santa Cruz, CA).

**Statistical analyses**

Statistical analyses were performed using Microsoft Excel or GraphPad v 9.1. Statistical significance for mouse survival was assessed by a Log-rank (Mantel Cox) test, body weight and fat content by restricted maximum likelihood method in a mixed-effects model for or one-way ANOVA and Dunnett's multiple comparisons test, glucose tolerance and rotarod by two-way ANOVA or the restricted maximum likelihood method in a mixed-effects model for and Dunnett's multiple comparisons test, inflammation and glumerulopathy score by two-way ANOVA and Tukey's multiple comparisons test, treadmill and grip strength by one-way ANOVA or unpaired two-tailed t test and Tukey’s or Dunnett's multiple comparisons test, water consumption by one-way ANOVA and Bonferroni's multiple comparisons test, by two-way ANOVA, Western blot by two-tailed unpaired t test.

**Supplemental Figure 1**

**Figure S1**: Western blot analysis of hippocampus lysate to demonstrate the inhibitory effect of lithium chloride on GSK3β demonstrated by serine 9 phosphorylation. The ratio of GSK3β phosphorylation to total GSK3β was significantly increased when 0.1 g LiCl/kg diet was administered to mice (P= 0.017, n=3).

**Supplemental Figure 2**

**Figure S2:** Lithium chloride administered at doses of 0.02 and 0.05 g/kg diet from 18 months of age (see arrow) did not affect lifespan of male (left) or female (right) C57Bl/6J mice (n=50). Statistical analyses by log-rank test.

**Supplemental Figure 3**

**Figure S3:** Lithium chloride administered at dose of 0.1 g/kg diet from 19 months of age (see arrow) did not affect lifespan female C57Bl/6J mice (n=43). Statistical analyses by log-rank test.

**Supplemental Figure 4**

**Figure S4:** Average water consumption of of male (left) and female (right) C3B6F1 mice during the lithium carbonate pre-experiment (P<0.0001, n=10). Error bars indicate Min to Max. Statistical analyses were performed using one-way ANOVA and Bonferroni's multiple comparisons test. **P < 0.01, ****P < 0.0001

**Supplemental Figure 5**

**Figure S5:** Effects of lithium administration (a) at doses of 0.02 and 0.05 g LiCl/kg diet on growth curve (n=13-28) and development of fat content (n=13-15) of C57Bl/6J females exposed to LiCl from 3 months of age (see arrow) during their first year of life and (b) at doses of 0.5 and 1.05 g LiCl/kg diet on body weight and fat content (n=6-12) of C57Bl/6J females exposed to LiCl from 22 months of age at age of 26-28 months (26-28 mo). Error bars indicate SEM. Statistical analyses were performed using the restricted maximum likelihood method in a mixed-effects model for (a), one-way ANOVA for (b) and Dunnett's multiple comparisons test.

**Supplemental Figure 6**

**Figure S6:** Effects of lithium administration (a) at doses of 0.02 and 0.05 g LiCl/kg diet on glucose tolerance (n=14-15) of C57Bl/6J females exposed to LiCl from 3 months of age (see arrow) during their first year of life and (b) at doses of 0.5 and 1.05 g LiCl/kg diet on glucose tolerance (n=6-12) of C57Bl/6J females exposed to LiCl from 22 months of age at age of 26-28 months (26-28 mo). Error bars indicate SEM. Statistical analyses were performed using two-way ANOVA for (a), the restricted maximum likelihood method in a mixed-effects model for (b) and Dunnett's multiple comparisons test.

**Supplemental Figure 7**

**Figure S7:** Effects of lithium chloride administration at doses of 0.5 and 1.05 g LiCl/kg diet on (a) rotarod performance of C57Bl/6J female mice, (b) grip strength and (c) treadmill performance of C57Bl/6J male (left) and female (right) mice exposed to LiCl from 22 months of age at age of 26-28 months. Error bars indicate SEM. Statistical analyses were performed using the restricted maximum likelihood method in a mixed-effects model for (a), one-way ANOVA for (b) and (c) and Tukey’s or Dunnett's multiple comparisons test.

**Supplemental Figure 8**

**Figure S8**: Effects of lithium carbonate administration at the dose of 1.0 g Li_2_CO_3_ /kg diet on (a) rotarod and (b) treadmill performance of C3B6F1 female mice treated from 14 months of age. Error bars indicate SEM. Statistical analyses were performed using two-way ANOVA for (a) and two-tailed unpaired t test for (b).
